# Endothelial tPA-dependent recruitment of microglia to vessels protects the blood-brain barrier after stroke

**DOI:** 10.1186/s12987-026-00801-w

**Published:** 2026-03-28

**Authors:** Tamara Etuzé, Louis Fosset, Denis Vivien, Benoit Denis Roussel, Eloise Lemarchand

**Affiliations:** 1 Physiopathology and Imaging of Neurological Disorders (PhIND), INSERM UMR-S U1237, Université de Caen Normandie, Normandie Université, Institut Blood and Brain @ Caen-Normandie, Cyceron, Boulevard Becquerel, Caen, 14074 France; 2 Department of Clinical Research, Caen-Normandie University Hospital, Centre Hospitalier Universitaire (CHU), Caen, France

**Keywords:** Stroke, Microglia, Endothelial cells, Tissue-type plasminogen activator (tPA), Blood-brain barrier

## Abstract

**Background:**

Ischemic stroke is one of the leading causes of death and disability worldwide and thrombolysis, with tissue-type plasminogen activator (tPA) or its mutants, remains the only pharmacological treatment available for the acute phase. In this study, we hypothesised that endothelial tPA plays a key role in modulating the microglial response and maintaining blood brain barrier (BBB) integrity after stroke.

**Methods:**

Using a mouse model with endothelial-specific deletion of tPA (VeCad^Cre^ – tPA^Flox^), combined with a thrombotic stroke model and high-resolution imaging, we investigated the effects of endothelial tPA on vascular inflammation and microglial activation.

**Results:**

Our results demonstrate that microglia-vessel contacts increase post-stroke. Notably, deletion of endothelial tPA reduces vascular cell adhesion molecule 1 (VCAM1) expression and is associated with decreased microglial activation and fewer microglia-vessel contacts. Interestingly, endothelial tPA deletion also leads to increased BBB permeability and a heightened risk of haemorrhagic transformation following stroke.

**Conclusions:**

Collectively, these findings indicate that endothelial tPA promotes microglial recruitment to blood vessels and plays a protective role in preserving BBB integrity after ischemic stroke.

**Supplementary Information:**

The online version contains supplementary material available at 10.1186/s12987-026-00801-w.

## Introduction

Ischemic stroke is one of the leading causes of death and disability worldwide [1]. Tissue damage after stroke is initially due to the arterial occlusion and the decrease in cerebral blood flow. Secondary damage can occur after stroke and is partially associated to an exacerbation of the inflammatory response, increased permeability of the blood-brain barrier (BBB) and haemorrhagic transformations [2]. Currently, thrombolysis (using tissue-type plasminogen activator; rtPA; or one of its mutants; tenecteplase) is the only pharmacological treatment available for the acute phase of ischemic stroke [3]. This is often combined with endovascular thrombectomy, to limit brain damage caused by cerebral artery occlusion. Although rtPA is beneficial due to its fibrinolytic action in the treatment of stroke, high levels of circulating rtPA administered during thrombolysis can exacerbate secondary brain damage, including increased BBB permeability and haemorrhagic transformation [4–6]. Additionally, preclinical studies have shown that endogenous tPA, particularly that produced by neurons, contributes to the deleterious effects of tPA on the brain parenchyma [7, 8].

Inflammation following stroke is well known to play a critical role in the progression of brain damage, with microglia being the first immune cell type to respond to disruption in brain homeostasis and to produce key immune mediators [2]. Recent evidence indicates that the interaction between microglia and blood vessels plays a crucial role in maintaining BBB integrity during disease, as well as in neurovascular coupling [9, 10]. Studies have shown that microglial P2RY12 is critical for interactions between microglia and BBB components [9, 11]. However, endothelial signalling leading to the recruitment of microglia to blood vessels remains unknown, in particular in the context of cerebrovascular diseases.

Here, we show that endothelial-derived tPA mediates microglia-vessel interactions during stroke, contributing to BBB integrity and dampening inflammatory responses. Using a mouse model with constitutive endothelial-specific deletion of tPA, we investigated its role in microglial activation and BBB permeability following stroke. Our results demonstrate that endothelial tPA is required for the recruitment of microglia to blood vessels after stroke and that its deletion exacerbates BBB disruption and promotes haemorrhagic transformation. Altogether, these findings indicate that endothelial tPA facilitates microglial recruitment to the vasculature, thereby supporting BBB protection in the context of stroke.

## Materials and methods

### Animals

All animal procedures adhered to the European Council Directives 2010/63/EU and were approved by the local ethical committee of Normandy (CENOMEXA). Experiments were performed according to the Animal Research: Reporting of In Vivo Experiments (ARRIVE) guidelines. All mice were housed in a room with automatically controlled temperature (21–25 °C), relative humidity (45–65%), and light-dark (12–12 h) cycles.

Twelve C57BL/6 male mice (Janvier Labs) and 39 VeCadCre: tPAlox male mice on a C57BL/6 background (Centre Universitaire de Ressources Biologiques, Caen, France), aged 12 to 16 weeks, were used. Endothelial specific tPA knockout mice (VE-Cre^ΔtPA^) were generated by crossing mice with exon 3 of the Plat gene flanked by loxP sites with VE-Cadherin-Cre mice (B6.FVB-Tg(Cdh5-cre)7Mlia/J; # 006137; kind gift from F. Millat, Institute of Radioprotection and Nuclear Safety in Fontenay-aux-Roses, France), to obtain VE-Cre^ΔtPA^mice and VE-Cre^WT^mice. The sample size was determined based on prior studies conducted with the same stroke model and to ensure adequate power to detect a pre-specified effect. Experiments and analysis were conducted in a blinded manner.

### Thrombotic stroke model

Animals were anesthetized with 5% isoflurane in a gas mixture of 70% nitrous oxide and 30% oxygen, placed in a stereotaxic device and maintained under anaesthesia (1,5 − 2% isoflurane; 70% N_2_O/30% O_2_). A small craniotomy was performed on the parietal bone to expose the M1-M2 segments of the middle cerebral artery (MCA). A Whatman filter paper strip soaked in freshly prepared AlCl_3_ solution (40%, Sigma-Aldrich) was placed on the intact dura mater to cover the bifurcation of MCA for 5 min [12]. AlCl₃ was used in order to improve MR imaging compared to FeCl₃, particularly to allow better visual discrimination of hemorrhagic transformation on T2-weighted images [13]. Sixteen mice were excluded due to inadequate arterial occlusion and absence of ischemic lesion.

For anti-VCAM1 (Vascular cell adhesion molecule 1) antibody treatment, mice received an intravenous injection of anti–VCAM-1 antibody (2.5 mg/kg, clone A(429), 553329, BD Pharmingen) or control isotype IgG (553926, BD Pharmingen), 30 min after induction of thrombotic stroke model [14, 15].

### Intrastriatal LPS injection

Mice were anesthetized with 5% isoflurane (in a gas mixture of 70% nitrous oxide and 30% oxygen) and placed in a stereotaxic device. A craniotomy was performed (coordinates: 0.5 mm anterior, 2.0 mm lateral, − 3 mm ventral to the bregma) and LPS was injected (0.5 µg per mouse; 0111:B4; Sigma-Aldrich, L’isle d’Abeau, France) into the striatum using a glass microneedle.

### Magnetic resonance imaging (MRI)

MRI experiments were conducted on a Pharmascan 7T (Bruker, Germany). Mice were deeply anesthetized with 5% isoflurane and maintained with 1.5-2% isoflurane in 30% O2 / 70% N2O during the MRI acquisitions. High-resolution T2-weighted images were acquired to assess ischemic brain damage using a multislice multiecho sequence with the following parameters: TE/TR 48.6 ms/3000 ms; slice thickness 0,5 mm; image size 256; average 2; field of view 17,92; scan time 3 min 12 s. T1-weighted images were acquired to evaluate gadolinium extravasation, with the following parameters: TE/TR 4.46 ms/15 ms; slice thickness: 0.5 mm; image size 256; average 3; field of view 17,92; scan time 4 min 1 sec 920 ms. T1-weighted acquisitions were performed both before and 5 min after gadolinium injection (Clariscan^TM^Gé, GE Healthcare SAS). Gradient echo-planar imaging T2*-weighted sequences were used to detect haemorrhagic transformation, with the following parameters: TE/TR 8.706 ms/500 ms; Slice Thickness 0,5 mm; Image size 256; average 2; Scan time 3 min 12 s. Lesion volumes, gadolinium extravasation and haemorrhagic volumes were determined using ImageJ^®^ software on coronal brain sections obtained with MRI. Haemorrhagic transformation (HT) was scored as follows: (0) no HT; [1] small HT; [2] HT.

### Tissue processing

Anesthetized mice were transcardially perfused with cold heparinized saline. Brains were removed, post-fixed for 24 h in 4% paraformaldehyde and cryoprotected in 20% sucrose in PBS for 48 h at 4 °C before freezing in Epredia^™^ Cryomatrix^™^ embedding medium (6769006, Thermo Scientific).

### Immunohistochemistry and immunofluorescence

Cryostat-cut sections (50 µm) were obtained and stored in freezing solution (ethylene glycol 30%, glycerol 20%, PBS 50%) at -20°C. Sections were washed in PBS and incubated overnight at 4°C with primary antibodies diluted in 0.25% Triton X-100. The following primary antibodies were used: rat anti-VCAM1 (1:200, 553330, BD Pharmingen), goat anti-PODXL (1:200, AF1556, R&Dsystems), rabbit anti-Iba1 (1:500, ab178847, Abcam), rat anti-CD68 (1:1000; ab53444, Abcam), chicken anti-GFAP (1:1000; ab4674, Abcam). Primary antibodies were revealed using F(ab’)_2_ fragments of donkey anti-rabbit linked to Cy3, anti-rat linked to Alexa 488 and anti-goat linked to Cy5 (1:500, Jackson ImmunoResearch, West Grove, USA). Sections were coverslipped with an antifade medium containing DAPI.

For analysis of neutrophil infiltration and pericyte-microglia interactions, cryostat-cut sections (10um) were obtained. Sections were washed in PBS and incubated overnight at 4°C with primary antibodies diluted in 0.25% Triton X-100. The following primary antibodies were used: rat anti-Ly6G (1:200, 127602, BioLegend), goat anti-PODXL (1:200, AF1556, R&Dsystems), rabbit anti-Iba1 (1:500, ab178847, Abcam), goat anti-CD13 (1:100; AF2335, R&Dsystems). Primary antibodies were revealed using F(ab’)_2_ fragments of donkey anti-rabbit linked to Cy3, anti-rat linked to Alexa 488 and anti-goat linked to Cy5 (1:500, Jackson ImmunoResearch, West Grove, USA). Sections were coverslipped with an antifade medium containing DAPI. For quantification of neutrophils, images were acquired using Olympus VS120 slide scanning system coupled with a Hamamatsu Orca Flash 4.0 camera. Three to four scans of the full sections were acquired at x20 magnification. For quantification of pericyte-microglia interactions, images were acquired in the peri-infarct area using a Leica THUNDER Imager Tissue microscope.

### Quantification of microglia morphology and microglia-vessel interactions

Confocal images from coronal brain sections of defined areas (4 to 6 z-stack) were captured using Leica SP8 laser scanning confocal microscope. Three-dimensional modelling was performed on z-stacked images using Imaris10. Microglia morphology was quantified using 3DMorph [16], a MATLAB-based script that semi-automatically quantifies individual microglial morphology from three-dimensional (3D) data.

For the quantification of VCAM1-positive vessels and CD68-positive microglia, binary masks were generated for each channel using ImageJ and the Image Calculator “AND” function was used to measure the percentage of PODXL area covered by VCAM1 or the percentage of microglia covered by CD68. Results are expressed as the percentage of VCAM1-positive vessels or the percentage of CD68 positive signal in microglia.

For the quantification of microglia-vessel interactions, binary masks were generated for PODXL + and Iba1 + signals using ImageJ. Podocalyxin was used to label vessels because of its consistent expression on the luminal surface of endothelial cells after stroke. The Image Calculator “AND” function was then used to measure the percentage of vessels (PODXL+) covered by microglia (Iba1+). Results are expressed as the percentage of Iba1-positive signal (microglia) in contact with blood vessels. To investigate microglia recruitment to blood vessels, we quantified the presence of microglia within a defined perivascular area corresponding to 2.9 μm around blood vessels (PODXL+). Based on microglial morphology, a radial distance of 2.9 μm from the vessel was defined to selectively include microglial somas in direct contact with the vessel. The Image Calculator “AND” function was then used to measure the percentage of the peri-vascular area that was positive for Iba1. Results are expressed as the percentage of Iba1-positive signal in the peri-vascular area. Similar analyses were performed for the quantification of microglia in contact with VCAM1-positive and VCAM1-negative vessels. Individual data points represent the average of measurements from each z-stack.

### Isolation of brain microvessels

Microvessels were isolated as previously described by Boulay et al., 2015 [17]. Brain cortices were placed in ice-cold HBSS containing HEPES (10mM), dissociated with a Dounce homogenizer and centrifuged at 2,000 g for 10 min at 4 °C. The pellet was resuspended in ice-cold HBSS-HEPES containing 18% dextran and centrifugation at 4,000 g for 15 min at 4 °C. The myelin layer and supernatant were discarded, and the pellet was resuspended in ice-cold HBSS-HEPES containing 1% bovine serum albumin (BSA) and filtered (20 μm pore size, hydrophilic nylon membrane, 47 mm diameter, Millipore, NY2004700). Microvessels were detached from the filter in HBSS-HEPES-BSA and centrifuged at 2,000 g for 10 min at 4 °C. The vessel pellet was resuspended in HBSS-HEPES-BSA, transferred to a 1.5mL Eppendorf tube and centrifuged at 8000 g for 10 min at 4 °C. The pellet was then resuspended and dissociated in 50µL RIPA lysis buffer (Thermo Scientific, 89900) and centrifuged at 12,000 g for 15 min at 4 °C. Protein concentration was determined using the BCA protein assay (Thermo Scientific, 23225).

### Plasminogen-casein Zymography

tPA enzymatic activity was assessed on 7.5% SDS-polyacrylamide gel containing bovine casein (10 mg/mL, ICN Biomedicals Inc., diluted in 1.5 M Tris buffer, pH 8.8) and 1 mg/mL human plasminogen (Lys-Type, Calbiochem-Millipore, 528185). Samples (8 µg/well) were separated by electrophoresis at 110 V at 4 °C for 3.75 h. Gels were washed for 1 h in 2.5% Triton X-100 followed by 30 min in H_2_O. Gels were incubated in buffer containing with 7.5 mg/mL glycine and 2.9 mg/mL EDTA for 2 h at 37 °C. Caseinolytic bands were visualized with Coomassie Blue staining.

### Statistical analysis

Statistical analysis and data representation were performed using GraphPad Prism 10 software. Data are represented as mean ± SD. Normality was assessed using D’Agostino & Pearson test and Shapiro-Wilk test and outliers using ROUT test. Transformations were applied where necessary. Data were analysed using two-tailed unpaired *t-*test, one-way ANOVA (followed by Sidak’s multiple comparisons post hoc test) or Mann-Whitney *U* test. Haemorrhagic transformation scores were analysed using Kruskal-Wallis test (followed by Dunnett’s multiple comparisons post hoc test). Differences were considered statistically significant when *p* < 0.05.

## Results

### Microglia are recruited to blood vessels following ischemic stroke

**Fig. 1:**
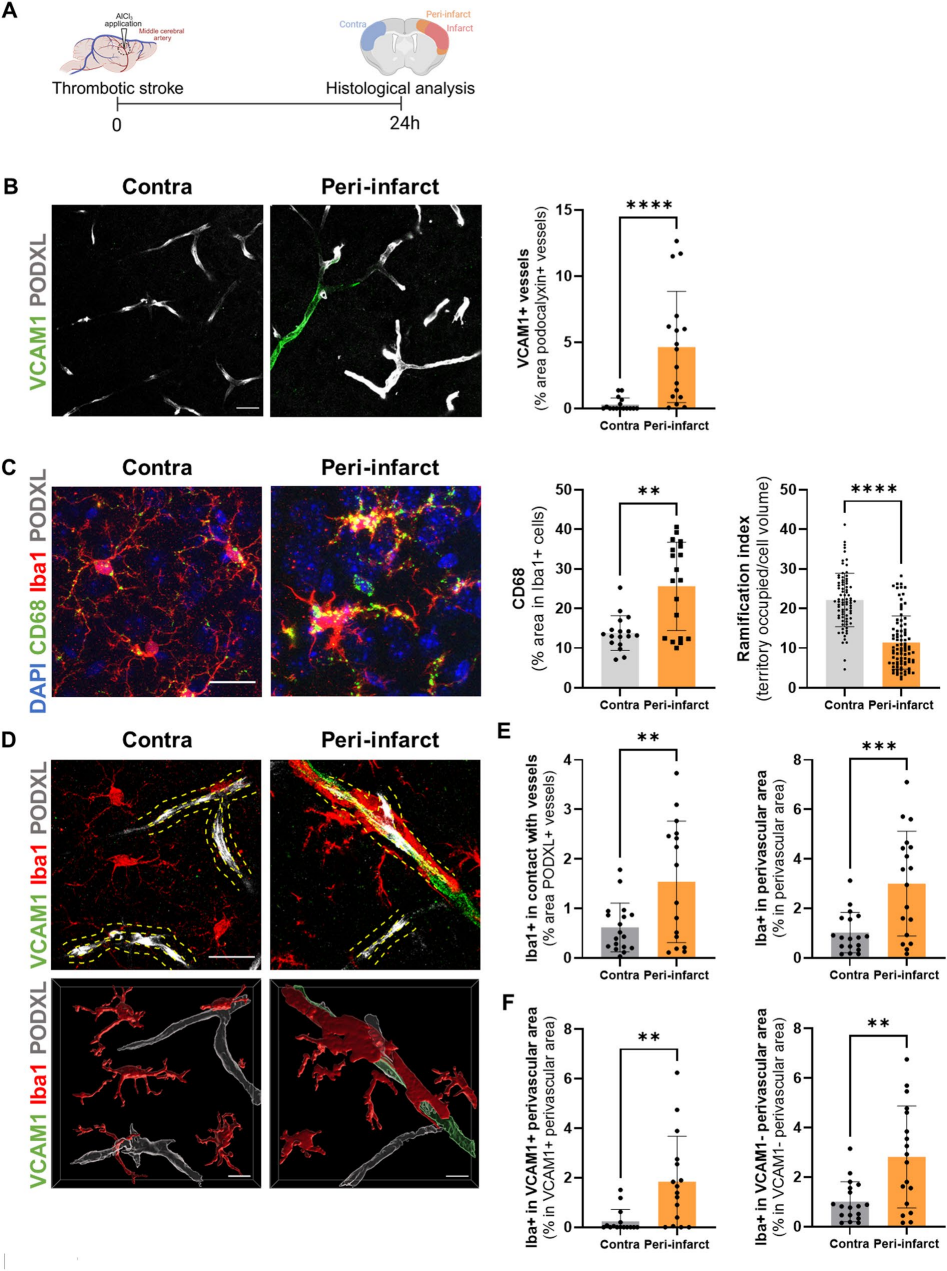
Vascular inflammation and microglial activation are associated with increased microglia-vessel interactions after stroke. (**A**) Schematic representation of the experimental design (created with BioRender.com). (**B**) Representative confocal images and quantification of VCAM1-positive vessels (podocalyxin, PODXL+) in contralateral and peri-infarct areas. Scale bar: 20 μm. (N = 4, n = 16 contralateral and N = 4, n = 17 peri-infarct). (**C**) Representative confocal images of vessels (PODXL), microglia (Iba1) and CD68 staining in the contralateral and peri-infarct areas, quantification of CD68 expression in Iba1-positive cells (N = 4, n = 17 contralateral, N = 4, n = 19 peri-infarct) and quantification of microglia ramification index (territory occupied/cell volume), (N = 4, n = 80 contralateral and N = 4, n = 89 peri-infarct). DAPI labels cell nuclei. Scale bar: 20 μm. (**D**) Representative confocal images of vessels (PODXL), microglia (Iba1) and VCAM1 staining in the contralateral and peri-infarct areas. Scale bar: 20 μm. (**E**) Quantification of microglial contacts with vessels and quantification of microglia present in peri-vessels areas (dotted yellow lines represent perivascular areas), (N = 4, n = 18 per group). (**F**) Quantification of microglia in VCAM1 positive and VCAM1 negative perivascular areas. (N = 4, n = 18 per group). Data were analysed using the Mann-Whitney U test (**B**, **F**) or a two-tailed unpaired t-test (**C**, **E**). **p < 0.01, ***p < 0.001, ****p < 0.0001. Data are shown as mean ± SD

To investigate the interaction between microglia and blood vessels after ischemic stroke, we first examined vascular inflammation and microglial responses in the peri-infarct and contralateral areas 24 h after induction of thrombotic stroke in mice (Fig. 1A). Vascular VCAM1 (an adhesion molecule upregulated upon endothelial activation and involved in leucocytes tethering to the endothelium) was significantly increased in the peri-infarct area compared with the contralateral side (15.67-fold increase compared to contralateral, *p* = 0.001; Fig. 1B). In addition, expression of the lysosomal marker CD68 was significantly increased in microglia (1.85-fold increase compared to contralateral, *p* = 0.0072; Fig. 1C) and microglial ramification complexity was significantly reduced in the peri-infarct area (1.94-fold decrease compared to contralateral, *p* < 0.0001; Fig. 1C), indicating enhanced microglial activation. These observations are consistent with prior reports using other stroke models [18, 19]. To further assess the interaction between inflamed vessels and microglia, we quantified microglial contacts with vessels. We observed a significant increase of microglial-vessel contacts in the peri-infarct area compared with the contralateral cortex (2.49-fold increase compared to contralateral, *p* = 0.0061; Fig. 1D-E). Moreover, microglial presence in defined perivascular areas was significantly elevated (2.93-fold increase compared to contralateral, *p* = 0.0008; Fig. 1E) around both VCAM1-positive vessels (7.57-fold increase compared to contralateral, *p* = 0.0022, Fig. 1F) and VCAM1-negative vessels (2.78-fold increase compared to contralateral, *p* = 0.0064; Fig. 1F). Altogether, our results demonstrate that stroke induces vascular inflammation in the peri-infarct region and promotes enhanced interactions between activated microglia and cerebral blood vessels.

### Microglial recruitment to blood vessels is mediated by endothelial tPA following ischemic stroke

**Fig. 2:**
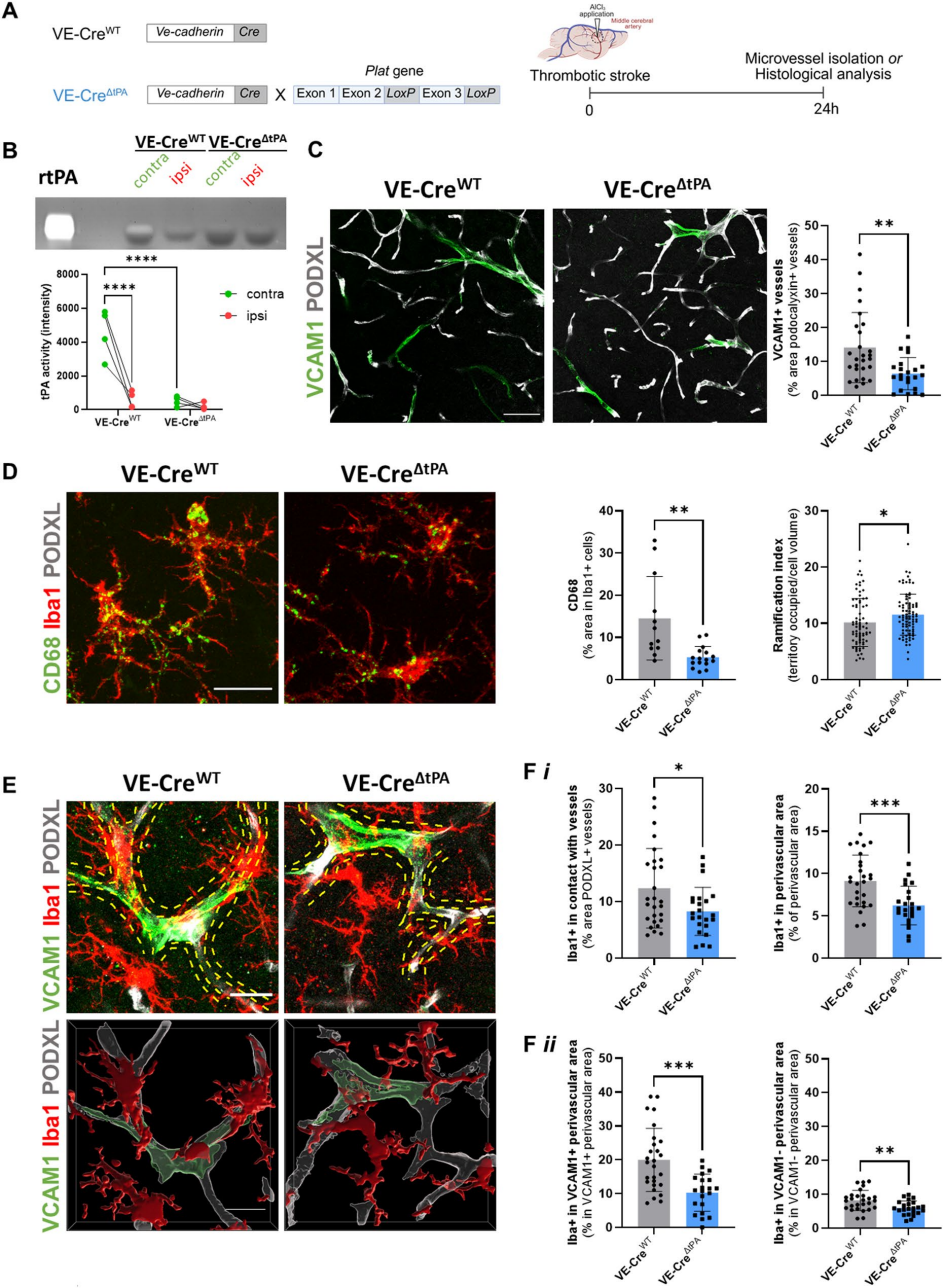
Endothelial tPA contributes to microglial recruitment to blood vessels after stroke. (**A**) Schematic representation of the experimental design (created with BioRender.com). (**B**) tPA activity in isolated microvessels from ipsilateral (ipsi) and contralateral (contra) cortex 24 h after stroke. (*N* = 4 per group, ****p < 0.0001, one-way ANOVA, uncropped gel in the supplementary figure). (**C**) Representative confocal images and quantification of VCAM1-positive vessels (podocalyxin, PODXL+) in the peri-infarct area. Scale bar: 50 μm. (*N* = 5, *n* = 27 VE-Cre^WT^ and *N* = 4, *n* = 23 VE-Cre^ΔtPA^). (**D**) Representative confocal images of vessels (PODXL), microglia (Iba1) and CD68 staining in the peri-infarct area (Scale bar: 30 μm), quantification of CD68 expression in Iba1-positive cells (*N* = 3, *n* = 12 VE-Cre^WT^ and *N* = 4, *n* = 16 VE-Cre^ΔtPA^) and quantification of microglia ramification index (territory occupied/cell volume), (*N* = 4, *n* = 74 VE-Cre^WT^ and *N* = 3, *n* = 86 VE-Cre^ΔtPA^). (**E**) Representative confocal images of vessels (PODXL), microglia (Iba1) and VCAM1 staining in the peri-infarct area (scale bar: 20 μm), (**F** *i*) quantification of microglial contacts with vessels and quantification of microglia present in perivascular areas. (**F** *ii*) Quantification of microglia in VCAM1 positive and VCAM1 negative perivascular areas (*N* = 5, *n* = 27 VE-Cre^WT^ and *N* = 4, *n* = 23 VE-Cre^ΔtPA^). Data were analysed using the Mann-Whitney *U* test (**C**) or a two-tailed unpaired *t* test (**D**, **F**). *p < 0.05; **p < 0.01; ***p < 0.001. Data are shown as mean ± SD

tPA is known to be released by endothelial cells following stroke as part of the endogenous fibrinolytic response [20]. Moreover, tPA has also been reported to modulate neuroinflammation and BBB integrity independently of its fibrinolytic activity [21]. We therefore hypothesized that endothelial tPA, released in response to ischemic stress, contributes to BBB disruption after stroke. Given recent evidence suggesting that microglia-vessel interactions play a role in maintaining BBB integrity, we further proposed that endothelial tPA facilitates the recruitment of microglia to blood vessels. To test this hypothesis, we generated a mouse model with a constitutive deletion of tPA specifically in endothelial cells [22, 23] (Fig. 2A). We first validated the deletion of tPA by isolating cortical microvessels from ipsilateral (red dots) and contralateral (green dots) cortices 24 h post-stroke in VE-Cre^WT^ and VE-Cre^ΔtPA^ mice (Fig. 2B). In VE-Cre^WT^ mice, endothelial tPA expression was significantly decreased in the ipsilateral cortex compared with contralateral side (7.70-fold decrease compared to contralateral, *p* < 0.0001), indicating stroke-induced tPA release. As expected, VE-Cre^ΔtPA^ mice showed nearly complete absence of endothelial tPA (8.97-fold decrease compared to VE-Cre^WT^ mice, *p* < 0.0001), confirming effective deletion. We next examined the consequences of endothelial tPA deletion on vascular inflammation and microglial activation. In VE-Cre^ΔtPA^ mice, VCAM1 expression in peri-infarct vessels was significantly reduced compared to VE-Cre^WT^ (2.21-fold decrease compared to VE-Cre^WT^ group, *p* = 0.0026; Fig. 2C). Microglial CD68 expression was also significantly decreased (2.73-fold decrease compared to VE-Cre^WT^ group, *p* = 0.0014; Fig. 2D) and microglial morphology indicated lower activation, with increased ramification index (1.13-fold increase compared to in VE-Cre^WT^ group, *p* = 0.0282; Fig. 2D). Furthermore, endothelial tPA deletion resulted in reduced microglial contacts with blood vessels (1.5-fold decrease compared to VE-Cre^WT^ group, *p* = 0.0212; Fig. 2E-F*i*) and fewer microglia were observed in the perivascular area (1.47-fold decrease compared to VE-Cre^WT^ group, *p* = 0.005; Fig. 2F*i*). This reduction occurred around both VCAM1-positive (2.73-fold decrease, *p* = 0.0001; Fig. 2F*ii*) and VCAM1-negative vessels (1.14-fold decrease, *p* = 0.0039; Fig. 2F*ii*). These alterations are specific to ischemic stress response, as we did not observe any changes in VCAM1 expression, microglial activation and microglia-vessel interactions between naïve VE-Cre^WT^ and VE-Cre^ΔtPA^ mice (Supplementary Fig. 1). As a decrease in VCAM1 expression was observed in VE-Cre^ΔtPA^ mice following stroke, we investigated its impact on neutrophil infiltration, as these cells are the first immune cells to enter the brain following stroke. However, no differences were observed in either infiltrating or vascular-associated neutrophils between VE-CreWT and VE-CreΔtPA mice (Supplementary Fig. 2). Altogether, these findings demonstrate that endothelial tPA promotes VCAM1 expression, microglial activation, and microglial recruitment to blood vessels in the peri-infarct area following stroke.

Astrocytes and pericytes are key components of the neurovascular unit and promote microglial recruitment to blood vessels under physiological conditions [9, 24]. Microglia also establish direct contacts with vessels through gaps between astrocytic endfeet [25]. We therefore analyzed microglial interactions with astrocytes and pericytes along the vasculature after stroke in VE-CreΔtPA and VE-CreWT mice. We observed a reduction in astrocytic and microglial contacts with vessels in VE-CreΔtPA, as well as reduced microglia–astrocyte interactions in the perivascular area. No differences were observed in the presence of GFAP-positive astrocytes in the perivascular area (Supplementary Fig. 3). These findings suggest an early BBB alteration 24 h after stroke, potentially involving astrocytic endfeet detachment and reduced microglial recruitment. In contrast, pericyte (CD13⁺) contacts with vessels and microglia–pericyte interactions were unchanged (Supplementary Fig. 4).

**Fig. 3:**
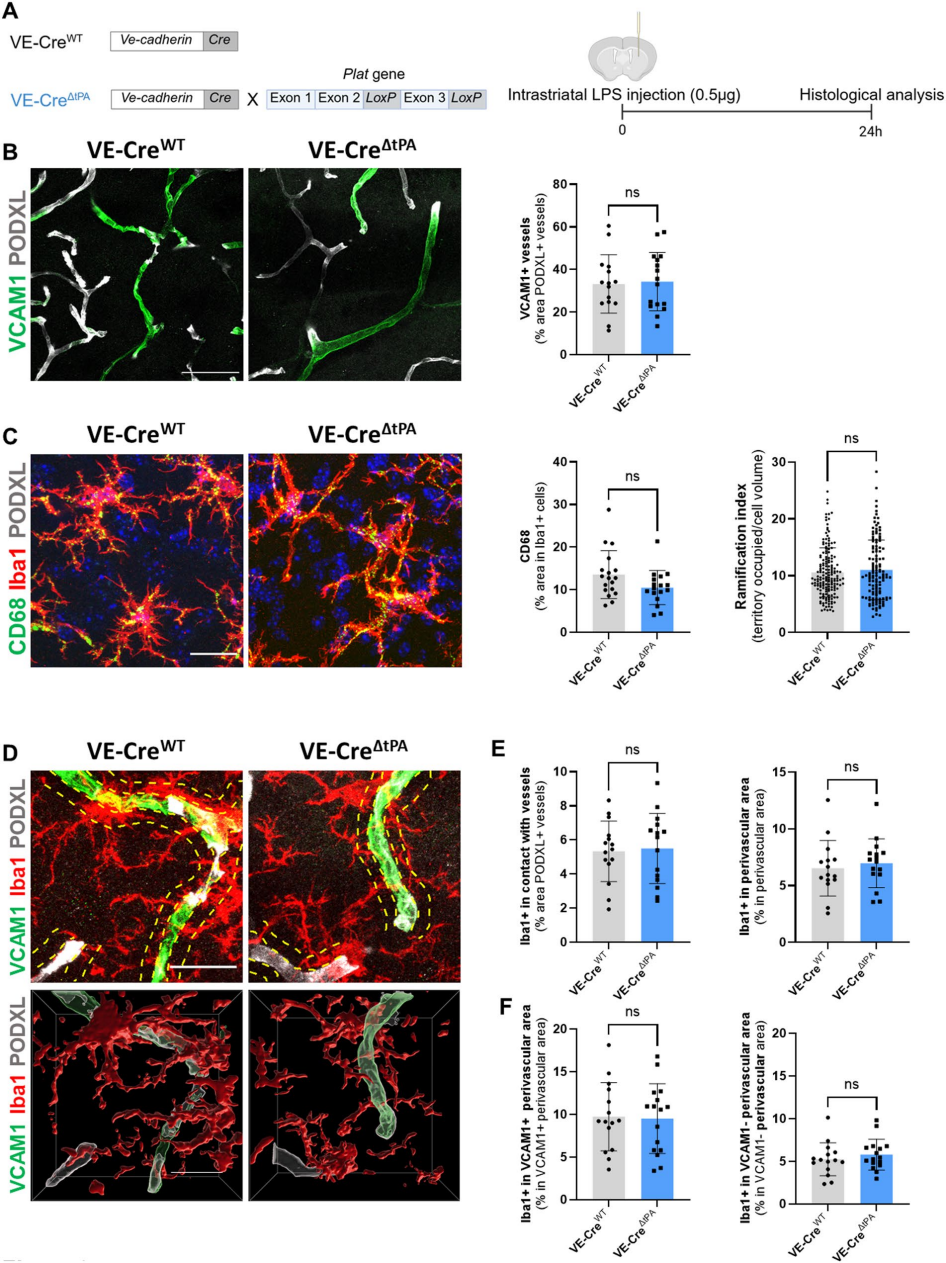
Endothelial tPA does not alter VCAM1 expression and microglial activation after LPS-induced neuroinflammation. (**A**) Schematic representation of the experimental design (created with BioRender.com). (**B**) Representative confocal images (DAPI labels cell nuclei, scale bar: 50 μm) and (**C**) quantification of VCAM1-positive vessels (podocalyxin, PODXL+) in the striatum 24 h after LPS (0.5 µg) injection. (N = 3, n = 15 VE-CreWT and N = 3, n = 16 VE-CreΔtPA) (**D**) Representative confocal images of vessels (PODXL), microglia (Iba1) and CD68 staining in the striatum (scale bar: 20 μm). (**E**) Quantification of CD68 expression in Iba1 positive cells (N = 3, n = 15 VE-CreWT and N = 3, n = 16 VE-CreΔtPA) and (**F**) measurement of microglial ramification index (territory occupied/cell volume) (N = 3, n = 169 VE-CreWT and N = 3, n = 129 VE-CreΔtPA). (**G**) Representative confocal images of vessels (PODXL), microglia (Iba1) and VCAM1 staining in the striatum (dotted yellow lines represent perivascular areas, scale bar: 20 μm) and quantification of (**H**) contact of microglia with vessels. Quantification of (**I**) microglia present in peri-vessels areas and (**J**) microglia in VCAM1 positive and (K) VCAM1 negative peri-vessels areas (N = 3, n = 15 VE-CreWT and N = 3, n = 16 VE-CreΔtPA). Data were analysed using two-tailed unpaired t-test. Data are shown as mean ± SD

To determine whether this effect of endothelial tPA is specific to ischemic stress or also occurs in other neuroinflammatory contexts, we used a model of inflammation induced by intrastriatal LPS injection (Fig. 3A). Unlike the stroke model, LPS-induced inflammation did not result in any differences between VE-Cre^ΔtPA^ and VE-Cre^WT^ mice in VCAM1 expression (Fig. 3B), microglial activation (Fig. 3C), or microglial recruitment to blood vessels (Fig. 3D-E). These results suggest that endothelial tPA plays a context-dependent role, specifically promoting microglial recruitment to the vasculature during ischemic injury but not during neuroinflammation.

### Microglial recruitment to blood vessels is independent of VCAM1

**Fig. 4:**
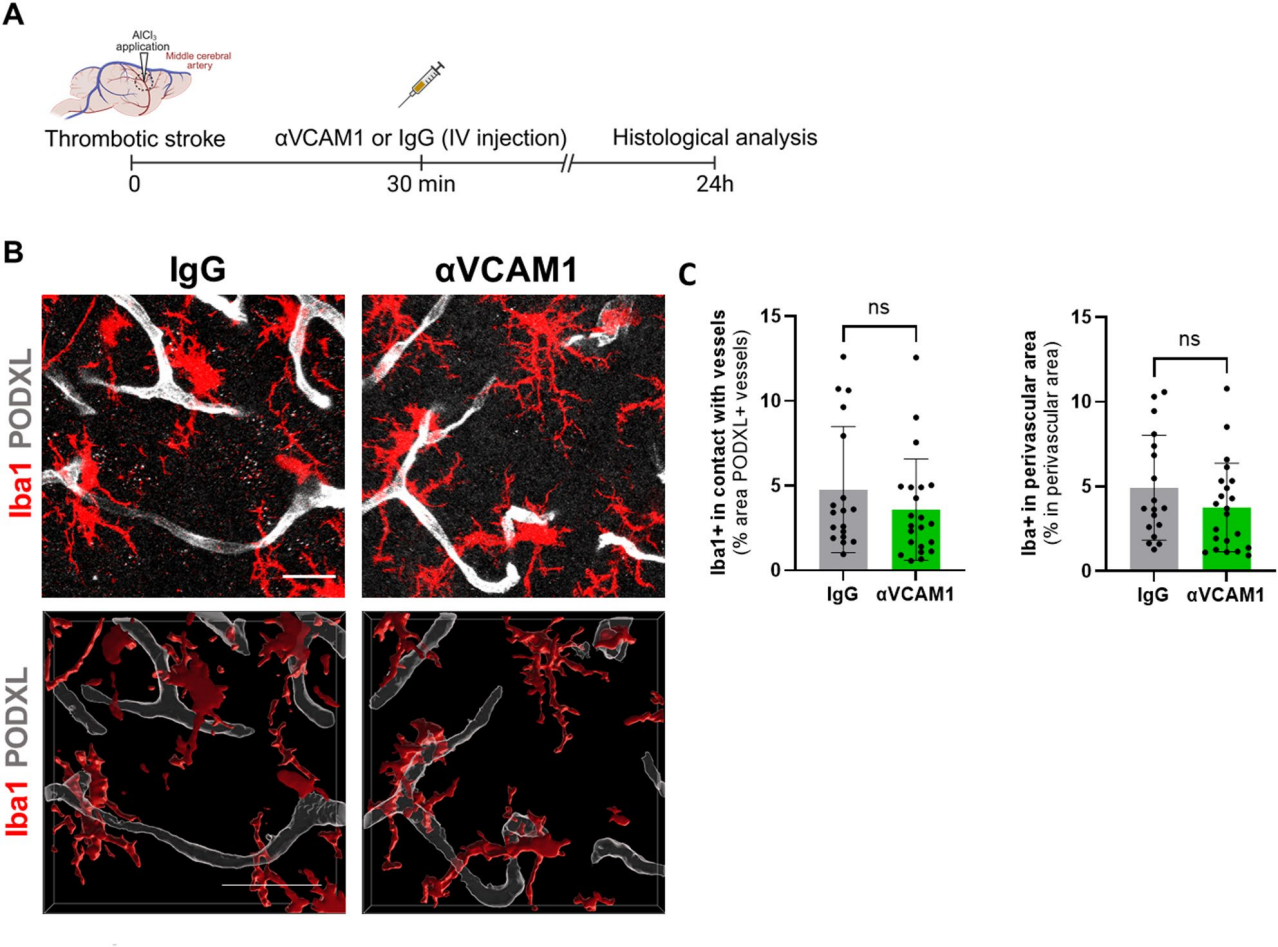
Microglial recruitment to blood vessels is independent of VCAM1.(**A**) Schematic representation of the experimental design (created with BioRender.com). (**B**) Representative confocal images of vessels (PODXL), microglia (Iba1), and VCAM1 staining in the peri-infarct area (scale bar: 20 μm). (**C**) Quantification of microglial contacts with vessels and quantification of microglia in perivascular areas (N = 4, n = 18 IgG and N = 4, n = 22 αVCAM1). Data were analysed using two-tailed unpaired t-test. Data are shown as mean ± SD

Our results show that VCAM1 expression is significantly upregulated following stroke (Fig. 1), an effect that is markedly attenuated in mice lacking endothelial tPA (Fig. 2). Based on this observation, we hypothesized that VCAM1 may mediate the recruitment of microglia to blood vessels. To test this hypothesis, we administered anti-VCAM1 blocking antibodies 30 min after stroke onset, and assessed microglia-vessel contacts 24 h later [14, 15] (Fig. 4A). Blocking VCAM1 did not significantly alter the percentage of microglial contacts with blood vessels or microglial presence in the perivascular space (Fig. 4B-C). These results suggest that, although VCAM1 is upregulated in endothelial cells following stroke, it does not play a major role in mediating microglial recruitment to blood vessels.

### Deletion of endothelial tPA exacerbates BBB permeability and haemorrhagic transformation after ischemic stroke

**Fig. 5:**
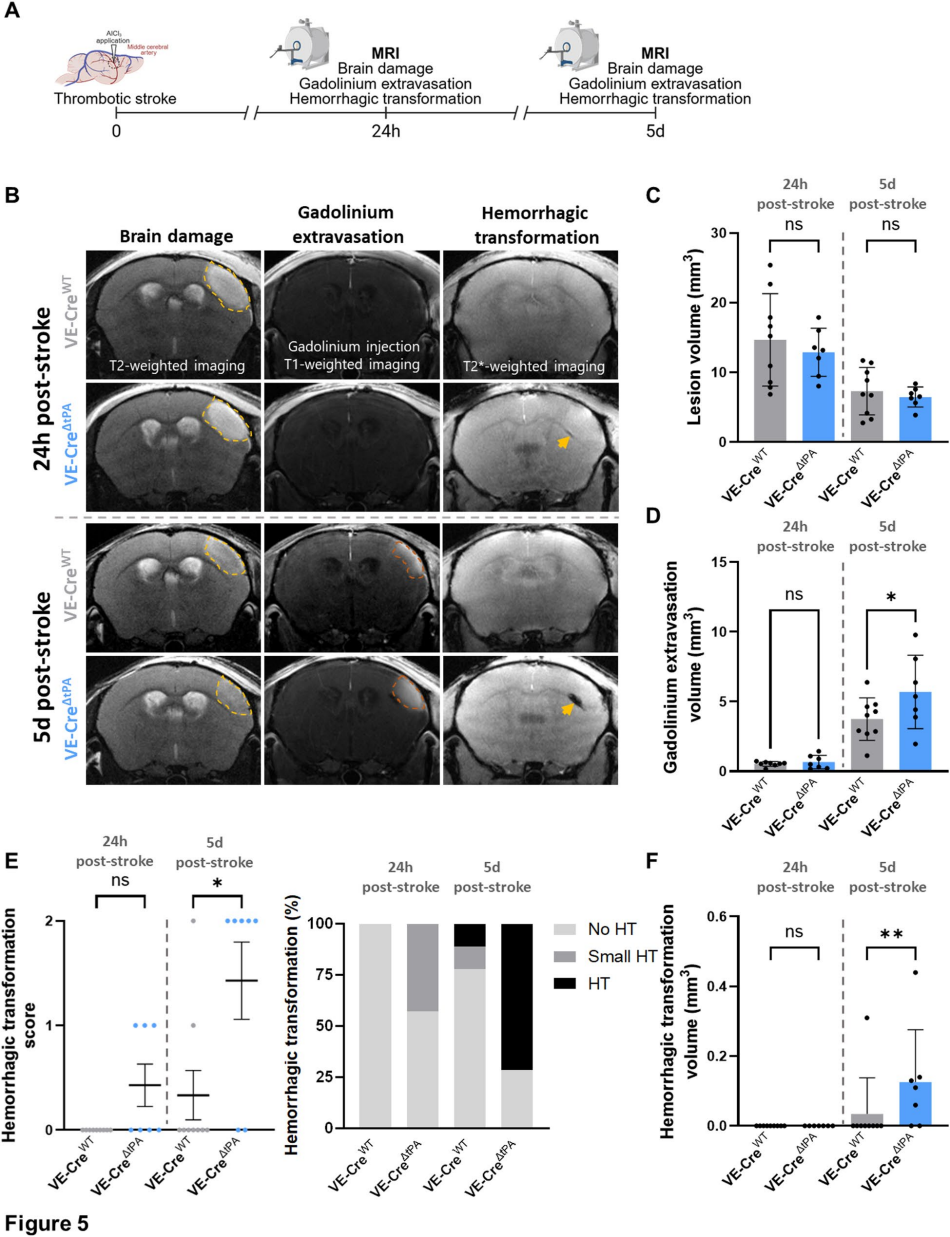
Endothelial tPA limits BBB permeability and haemorrhagic transformation 5 days after stroke. (**A**) Schematic representation of the experimental design (created with BioRender.com). (**B**) Representative T2-weighted, T1-weighted, and T2*-weighted images at 24 h and 5 days after stroke. Oedema is delineated by a discontinuous yellow line and haemorrhagic transformation (HT) is indicated by yellow arrow. Quantification of (**C**) lesion volumes and (**D**) gadolinium extravasation volumes. (N = 9 VE-CreWT and N = 7 VE-CreΔtPA, *p < 0.05, ordinary one-way ANOVA.) (**E**) HT score (no haemorrhagic transformation (0), small haemorrhagic transformation (1), haemorrhagic transformation (2)), distribution of HT score and (**F**) HT volume. (N = 9 VE-CreWT and N = 7 VE-CreΔtPA, *p < 0.05, **p < 0.01, Kruskal-Wallis test). Data are shown as mean ± SD

Given the established role of microglial interactions with blood vessels in maintaining BBB integrity [10, 11], we examined the consequences of endothelial tPA deletion, and the associated disruption of microglial-vessel interactions, on stroke outcome. To this end, we conducted longitudinal MRI to monitor lesion volume, gadolinium extravasation, and haemorrhagic transformation from 24 h to 5 days post-stroke (Fig. 5A). Lesion volumes did not significantly differ between VE-Cre^WT^ and VE-Cre^ΔtPA^ mice at either 24 h (14.7mm^3^ *versus* 12.9 mm^3^, *p* = 0.6670) or 5 days (7.3 mm^3^ *versus* 6.5 mm^3^, *p* = 0.9129) after stroke (Fig. 5B-C). In contrast, gadolinium extravasation was significantly increased in VE-Cre^ΔtPA^ mice compared to VE-Cre^WT^ 5 days after stroke (5.70mm^3^ *versus* 3.75mm^3^ in, *p* = 0.0330; Fig. 5D). Furthermore, haemorrhagic transformation was significantly more pronounced in VE-Cre^ΔtPA^ mice, as shown by increased scores and volumes (0.13mm^3^ *versus* 0.03mm^3^, *p* = 0.0080; Figs. 5E-F). These results demonstrate that endothelial tPA plays a critical role in preserving BBB integrity and limiting haemorrhagic transformation after stroke. Together, these findings suggest that endothelial tPA supports neurovascular protection, at least in part by facilitating microglia–vessel interactions.

## Discussion

Although rtPA remains one of the only approved pharmacological treatments for acute ischemic stroke due to its fibrinolytic activity, its use has been associated with adverse effects such as increased BBB permeability and heightened risk of haemorrhagic transformation [26]. These deleterious effects are partly attributed to rtPA-induced inflammatory responses [4, 6]. However, the role of endogenous tPA, particularly that derived from endothelial cells, in modulating inflammation and microglial activation remains less well understood. In this study, we investigated the specific contribution of endothelial-derived tPA to microglial responses and its impact on BBB permeability and haemorrhagic transformation following stroke.

We show that endothelial tPA plays a crucial role in recruiting microglia to blood vessels within the peri-infarct region during the early phase of ischemic stroke. This aligns with previous studies reporting that microglial P2RY12 is essential for microglial migration and microglia-vessel interaction in contexts such as laser injury, neurovascular coupling and cerebrovascular disease [9, 11, 27]. Microglial migration toward blood vessels has also been observed in response to systemic inflammation, driven by endothelial CCL5 release, leading to microglial expression of Claudin-5, which helps preserve BBB integrity during early inflammation [28]. However, in our study, endothelial tPA did not influence microglia-vessel interactions in a model of neuroinflammation, suggesting that its effect is specific to ischemic stress. We previously reported that endothelial tPA is rapidly released after stroke to facilitate endogenous fibrinolysis and support neurovascular coupling [22, 23]. The current findings suggest that endothelial tPA also promotes microglial recruitment to blood vessels in response to ischemia. Notably, this protective mechanism appears distinct from the effects of circulating tPA. In our model, endothelial tPA deletion does not affect plasma tPA levels [17], indicating that microglial recruitment to blood vessels is independent of circulating tPA. Moreover, the discrepancy between the protective effects of endothelial tPA and the pro-haemorrhagic actions of rtPA may be attributable to the supraphysiological doses of rtPA used in thrombolytic therapy compared to the localized, regulated release of endothelial tPA.

Our results indicate that microglial phagocytic activity is decreased in VE-Cre^ΔtPA^ following stroke. We also observed an increase in microglial ramification index, consistent with reduced microglial activation in VE-Cre^ΔtPA^ after stroke. Beyond its thrombolytic role, tPA has been reported to interact with microglia through several receptors. Interactions with LRP1 and Annexin II have been associated with alterations in microglial migration and activation, as well as with the release of inflammatory mediators and matrix metalloproteinases [21, 29]. tPA has also been suggested to modulate NMDA receptor signaling pathways in macrophages, potentially influencing the inflammatory response [30]. In this context, tPA may act as a cytokine promoting microglial activation. Moreover, tPA release can promote MMP9 activation, leading to extracellular matrix degradation; thus, this mechanism could also contribute to microglial recruitment to blood vessels [31]. However, these effects appear to be context-dependent, as we did not observe any alteration in microglial recruitment in a neuroinflammatory model. One limitation of our study is that the precise mechanisms through which tPA regulates microglial recruitment remain to be elucidated. Nevertheless, our findings demonstrate a critical role for endothelial tPA in regulating microglial recruitment to blood vessels following stroke.

Consistent with prior literature demonstrating the role of microglia in maintaining BBB integrity [11, 27], we observed that deletion of endothelial tPA exacerbates BBB permeability and increases the incidence of haemorrhagic transformation after stroke. Since endothelial tPA deletion was associated with reduced VCAM1 expression, we tested whether VCAM1 signalling contributes to microglial recruitment. However, VCAM1 blockade did not alter microglial–vessel interactions, indicating that this recruitment is VCAM1-independent and may not rely on classical endothelial activation pathways.

Our findings identify endothelial tPA as a critical regulator of microglia-endothelial interactions following stroke. By facilitating microglial recruitment to blood vessels, endothelial tPA supports BBB integrity and reduces the risk of haemorrhagic transformation, highlighting a previously underappreciated protective role of endogenous tPA in the neurovascular response to stroke.

## Supplementary Information

Below is the link to the electronic supplementary material.

Supplementary Material 1

## Data Availability

The data that support the findings of this study are available from the corresponding authors upon reasonable request.

## Abbreviations

ARRIVE: Animal Research: Reporting of in Vivo Experiments
BBB: Blood-brain barrier
BCA: Bicinchoninic acid
BSA: Bovine serum albumin
CBF: Cerebral blood flow
HT: Hemorrhagic transformation
LRP1: Low-Density Lipoprotein Receptor–Related Protein-1
MCA: Middle cerebral artery
MRI: Magnetic resonance imaging
P2RY12: Purinergic receptor P2Y12
tPA: Tissue-type plasminogen activator
VCAM1: Vascular cell adhesion molecule 1
