# Supplementary material for: Endothelial tPA-dependent recruitment of microglia to vessels protects the blood-brain barrier after stroke
Source: Fluids Barriers CNS. 2026 Mar 28;23:67. doi: 10.1186/s12987-026-00801-w (PMC13151305; doi:10.1186/s12987-026-00801-w)
Supplement: Supplementary file 1 — Supplementary Material 1 [file 12987_2026_801_MOESM1_ESM.pdf]

Supplementary Figures

Endothelial tPA-dependent recruitment of microglia to vessels protects the blood-brain barrier after stroke

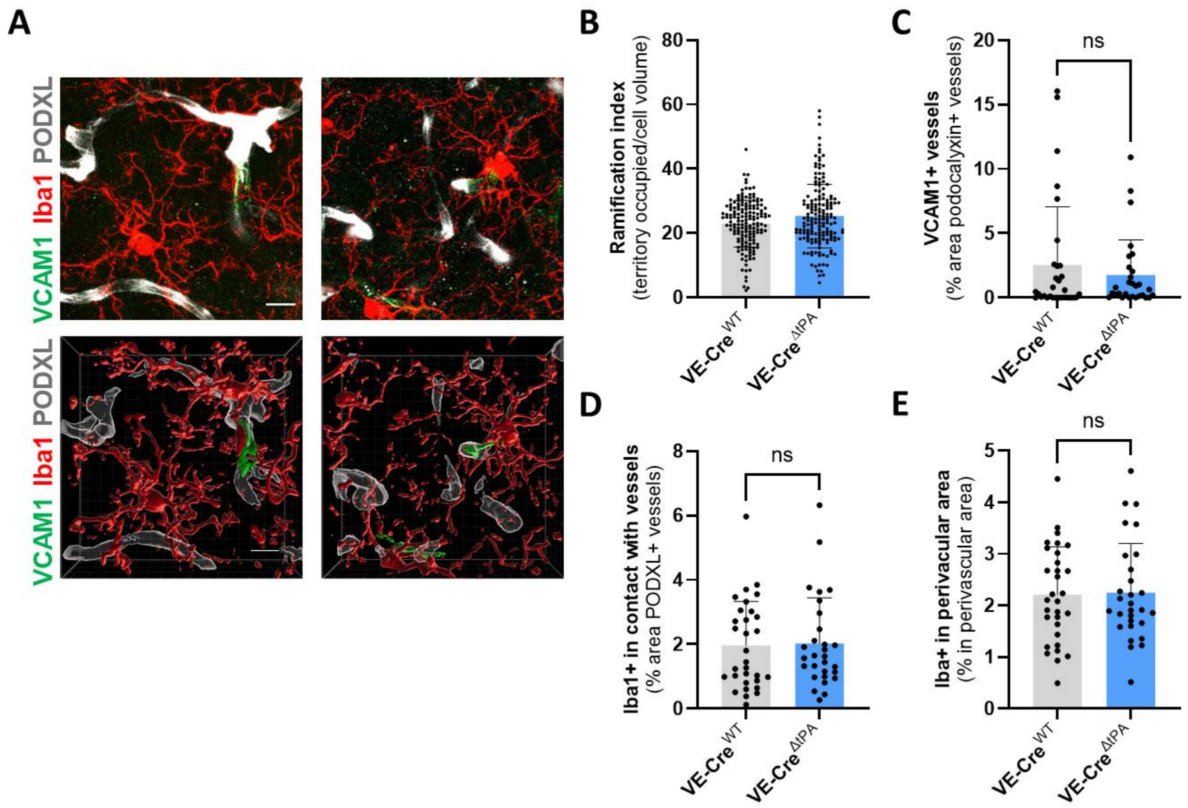

Supplementary Figure 1: Constitutive deletion of endothelial tPA does not alter microglial activation, VCAM1 expression, and microglial contact with vessels in naïve mice.

(A) Representative confocal images of vessels (PODXL), microglia (Iba1) and VCAM1 staining in the peri-infarct area (scale bar: 10 µm). (B) Quantification of microglia ramification index (territory occupied/cell volume), (N=4, n=161 VE-Cre^{WT} and N=4, n=165 VE-Cre$^{\Delta tPA}$). (C) Quantification of VCAM1-positive vessels (podocalyxin, PODXL+) in the perivascular area. (D) Quantification of contact of microglia with vessels and (E) quantification of microglia present in perivascular areas. (N=4, n=29 VE-Cre^{WT} and N=4, n=31 VE-Cre$^{\Delta tPA}$). Data were analysed using a two-tailed unpaired t-test (E) or using the Mann-Whitney test (B, C, D). Data are shown as mean ±SD.

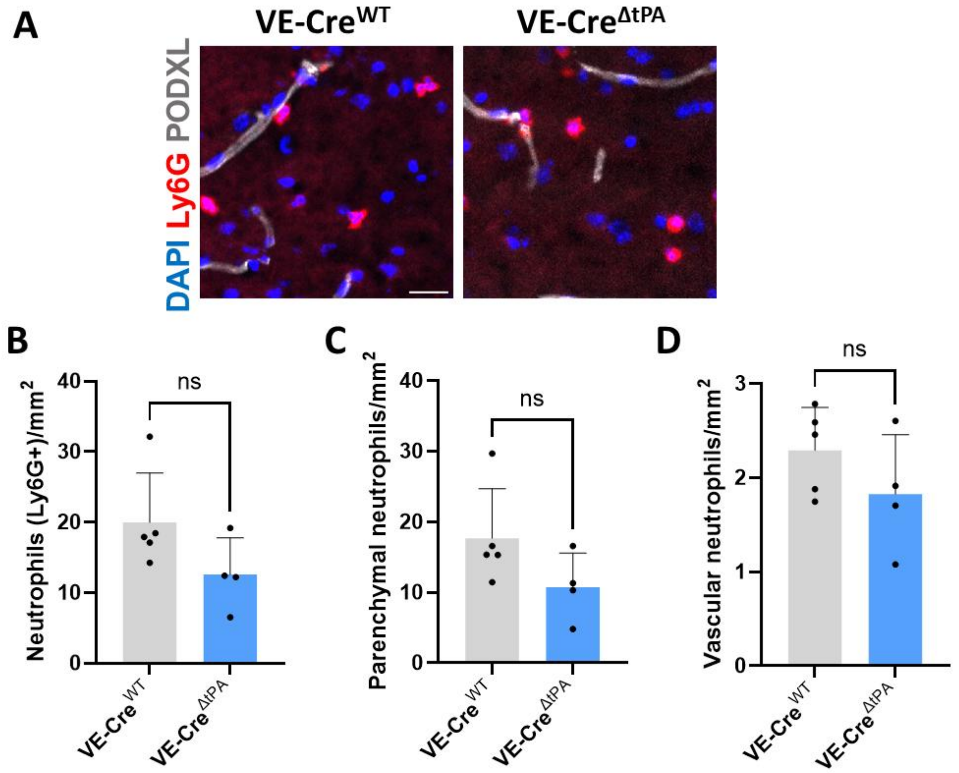

Supplementary Figure 2: Endothelial tPA does not influence neutrophil infiltration 24 hours after stroke

(A) Representative images (DAPI labels cell nuclei, scale bar: 20 µm) and (B) quantification of neutrophils (Ly6G+ cells) within the infarct 24h after stroke. (N=4-5/group) (C) Quantification of neutrophils present in the parenchyma and (D) associated with vessels (Ly6G+ and PODXL+) (N=5-4/group). Data were analysed using the Mann-Whitney test. Data are shown as mean ±SD.

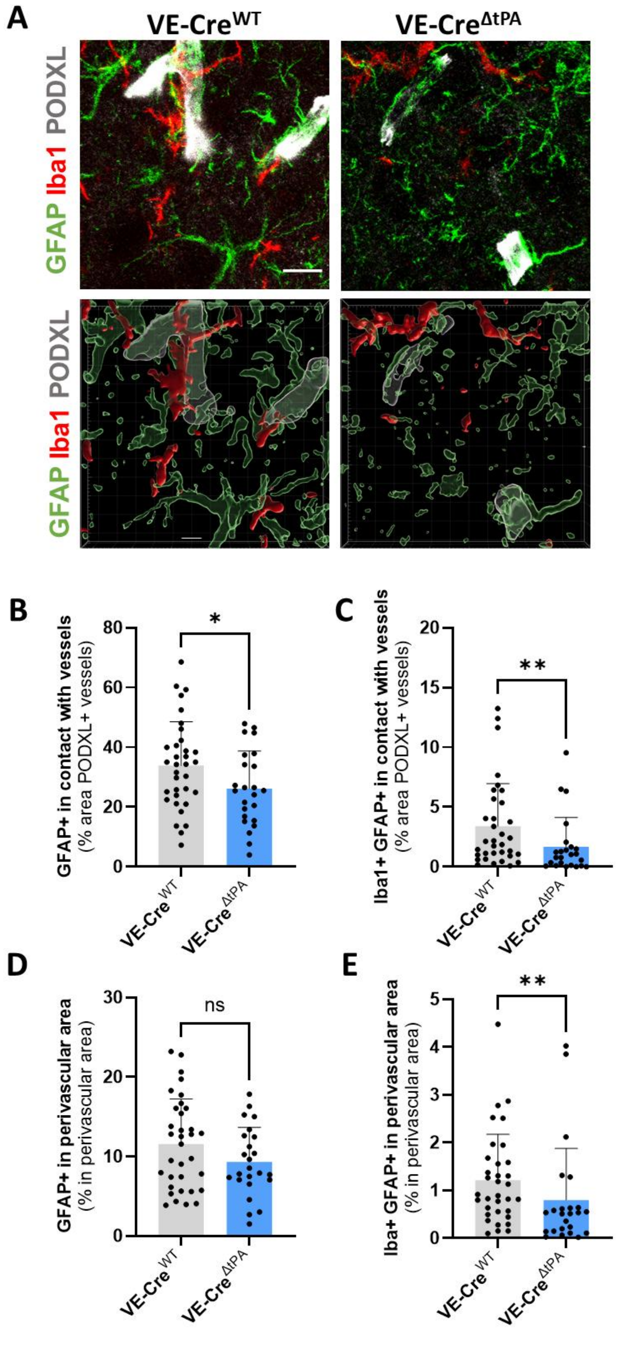

Supplementary Figure 3: Deletion of endothelial tPA alters microglia and astrocyte interactions with vessels 24h after stroke.

(A) Representative confocal images of vessels (PODXL), microglia (Iba1) and astrocytes (GFAP) in the peri-infarct area (Scale bar: 10 µm). (B) Quantification of contact of astrocyte-vessel contacts (GFAP+/PODXL+). (C) Quantification of microglia–astrocyte–vessel contacts ($GFAP^+$/$Iba1^+$/$PODXL^+$). (D) Quantification of astrocytes in perivascular areas. (E) Quantification of microglia-astrocyte contacts in perivascular areas (N=5, n=34 VE-CreWT and N=4, n=24 VE-CreΔtPA). Data were analysed using a two-tailed unpaired t-test (B, D) or the Mann-Whitney test (C, E). *p<0.05; **p<0.01, ****p<0.0001. Data are shown as mean ±SD.

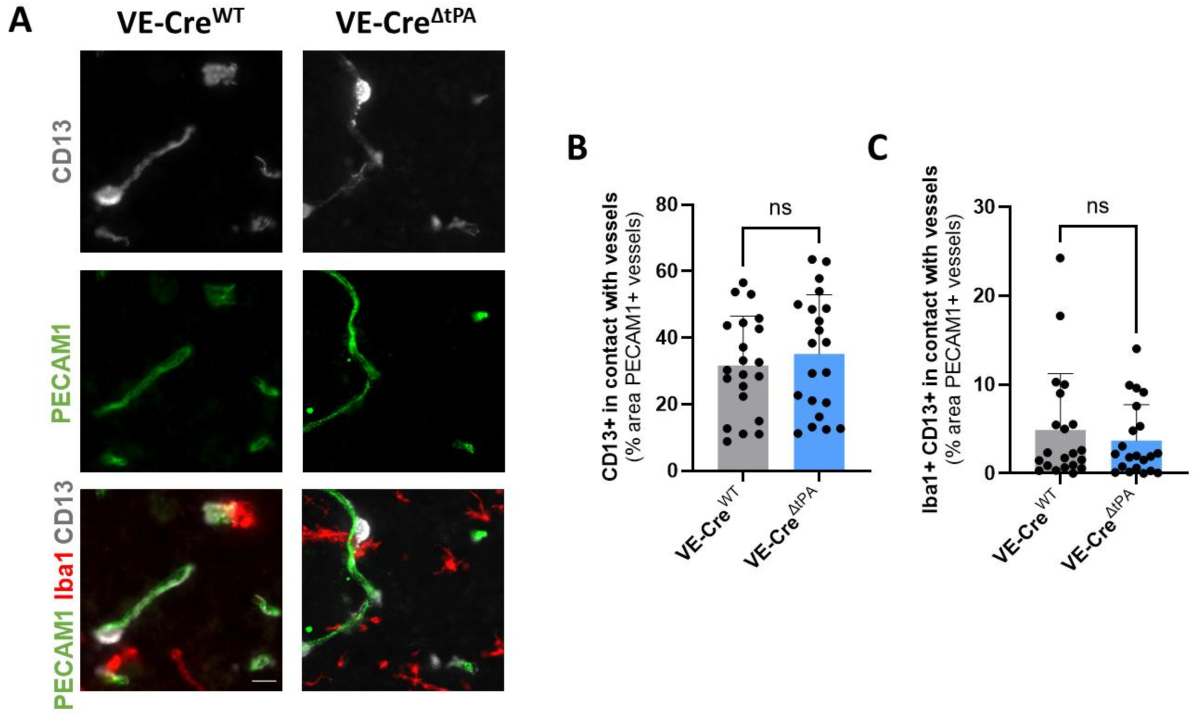

Supplementary Figure 4: Deletion of endothelial tPA does not affect pericyte-microglia-vessel interactions 24h after stroke.

(A) Representative images of vessels (PODXL), microglia (Iba1) and pericytes (CD13) in the peri-infarct area (scale bar: 10 µm). (B) Quantification of pericyte-vessel contacts (CD13+/PODXL+). (C) Quantification of microglia–pericyte–vessel contacts ($CD13^{+}/Iba1^{+}/PODXL^{+}$) (N=5, n=21 VE-CreWT and N=4, n=21 VE-CreΔtPA). Data were analysed using a two-tailed unpaired t-test (B) or the Mann-Whitney test (C). Data are shown as mean ±SD.

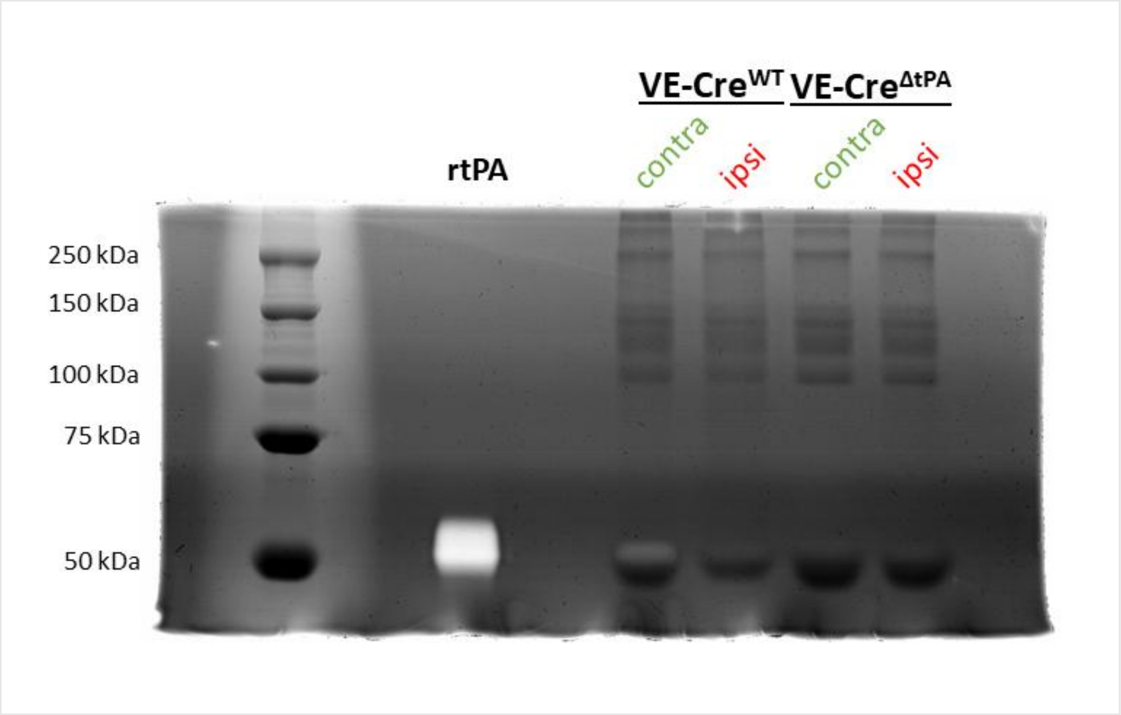

Supplementary Figure 5: Uncropped tPA zymography gel appearing in Figure 2B.
